# Evaluation of OwnHealth^®^: a telephone based care management service for long-term conditions in the West Midlands

**Published:** 2012-06-15

**Authors:** Iain Donald McNeil, Liv Nymark

**Affiliations:** Pfizer Health Solutions, United Kingdom; Pfizer Health Solutions, United Kingdom

**Keywords:** evaluation, self-care, long-term, scale, savings

## Abstract

OwnHealth^®^ is a proactive telephone based self care programme for people with long-term conditions. The service, operating continuously since 2006, promotes self efficacy through education and supporting health related behaviour changes has resulted in reductions in unscheduled secondary care and associated costs, high levels of user satisfactions and improved clinical outcomes. The service has been delivered to 17,569 members in the West Midlands since it started with 8486 people enrolled and receiving care as of 1st October 2011. It is the largest proactive long-term condition telephone based care management service in the UK.

**Cost effectiveness:**

A retrospective study conducted in 2011 asked if the OwnHealth^®^ programme had any effect on reducing costs by preventing spells of unscheduled secondary care. A data mining model based on cluster technique was used to select patients from the intervention and the matched control group. This resulted in the selection of 8400 cases: 4200 in the OwnHealth^®^ intervention and 4200 cases in control group. These patients all met the inclusion criteria for enrolment in the service and were matched based on demographic data and baseline clinical metrics. Data were collected on the number of ‘spells’ each patient had in hospital from the Commissioning Data Sets (CDS), which cover in-patient activity, out-patient appointments and A&E attendances. The cost of each spell was calculated using national tariffs. A reduction in spells per year with the OwnHealth^®^ programme was demonstrated for 7 out of 10 of the long-term conditions included in the study. The reductions in spells were statistically significant. The cost in the patients group enrolled in the OwnHealth^®^ programme was lower than in the control group for all disease areas. The reduction in cost was statistically significant for all indications apart from stroke and transient ischaemic attack. It is estimated that based on this financial model and taking into account service delivery costs, that the commissioners would be able to release at least £2 million per year and make this available elsewhere.

**Member satisfaction:**

The results of surveys of member‘s satisfaction with the service provided, and their perceived impact of the service upon them indicate high levels of satisfaction with both the service and care management. From the outset the member’s satisfaction levels were high; this high-level of satisfaction and the impact on an individual’s ability to self care has been maintained throughout the term of the service.

**Clinical effectiveness:**

The self management of long-term conditions which is facilitated by the programme has also resulted in improved clinical outcomes. An independent comparison study involving patients enrolled in the programme with poorly controlled diabetes, showed that the OwnHealth^®^ intervention significantly improved clinical measures of disease progression compared to the matched control group sourced from patients on the General Practice Research Database (GPRD).

**Summary:**

The Birmingham OwnHealth^®^ programme demonstrates cost savings, very high levels of customer satisfaction and sustained clinical and behavioural changes.

